# Hypermethylated *CDO1* and *ZNF454* in Cytological Specimens as Screening Biomarkers for Endometrial Cancer

**DOI:** 10.3389/fonc.2022.714663

**Published:** 2022-04-28

**Authors:** Lei Wang, Lanlan Dong, Jun Xu, Lin Guo, Yiran Wang, Kangkang Wan, Wei Jing, Lanbo Zhao, Xue Feng, Kailu Zhang, Miao Guo, Yuliang Zou, Lianglu Zhang, Qiling Li

**Affiliations:** ^1^ Department of Obstetrics and Gynecology, The First Affiliated Hospital of Xi’an Jiaotong University, Xi’an, China; ^2^ Wuhan ammunition Life-Tech Co., Ltd., Wuhan, China; ^3^ Department of Obstetrics and Gynecology, Tangdu Hospital, the Fourth Military Medical University, Xi’an, China; ^4^ Department of Gynecologic Oncology, Shaanxi Provincial Cancer Hospital, Xi’an, China; ^5^ State Key Laboratory of Virology, Department of Biochemistry and Molecular Biology, College of Life Sciences, Wuhan University, Wuhan, China

**Keywords:** cysteine dioxygenase type 1 (*CDO1*), endometrial cancer (EC), endometrial cytology test (ECT), zinc finger protein 454 (*ZNF454*), DNA methylation, screening, cytology

## Abstract

We aimed to estimate the diagnostic value of DNA methylation levels in cytological samples of endometrial cancer (EC) and atypical hyperplasia (AH). Two hypermethylated genes, namely, cysteine dioxygenase type 1 (*CDO1*) and zinc finger protein 454 (*ZNF454*), in patients with EC were identified from The Cancer Genome Atlas database. In 103 endometrial histological specimens (the training set), the methylation levels of candidate genes were verified by quantitative methylation-specific polymerase chain reaction (qMSP). The methylation levels of another 120 cytological specimens (the testing set) were evaluated. Sensitivity (Se), specificity (Sp), accuracy, and area under the curve (AUC) were determined, with diagnosis verified by histopathological results. *CDO1* and *ZNF454* verified hypermethylation in histological specimens of patients with EC and AH compared with those with benign and normal endometrium (*P* < 0.001). In cytological specimens, hypermethylated *CDO1* showed 86.36% Se and 90.79% Sp with the cutoff value of 6.0 to distinguish between malignant and benign groups; *ZNF454* showed 79.55% Se and 93.42% Sp with the cutoff value of 7.1. When the two genes were combined, Se increased to 90.91% and Sp was 86.84%. AUC reached 0.931 (95% CI: 0.885–0.976). The diagnostic accuracy with cytology had no significant difference with endometrial tissue (*P* = 0.847 for *CDO1*, *P* = 0.108 for *ZNF454*, and *P* = 0.665 for their combination). Hypermethylated *CDO1* and *ZNF454* in endometrial cytology showed high Se, Sp, and AUC to detect EC and AH. Methylation analysis of endometrial cytology is promising biomarker for the screening of EC and AH.

## Introduction

Among female reproductive tract cancers, endometrial cancer (EC) is the most prevalent in developed countries (1). The 5-year survival rate of early diagnosed cases (stage I and stage II) ranges from approximately 96% to 74% (2, 3). However, approximately 15% of ECs are diagnosed in stages III–IV with a very poor prognosis (4). The risk of developing EC from endometrial hyperplasia without atypia is around 3% and increases up to 23% in endometrial atypical hyperplasia (AH) (5). It is very important to screen AH and early EC in women at high risks (6).

There are no highly sensitive and standardized screening methods for EC (7). Transvaginal ultrasound is a non-invasive diagnostic test but the cutoff value for endometrial thickness remains unclear. With a low specificity (Sp) of 24.3%–74%, transvaginal ultrasound cannot reliably identify malignant lesions (8, 9). Although traditional dilation and curettage (D&C) is relatively effective and hysteroscopic biopsy can be performed to make a definite diagnosis, these are invasive procedures to obtain endometrial tissues (10, 11). Hysteroscopic biopsy or D&C collects limited amount of endometrial tissue, which results in difficulty and disagreement for pathological diagnosis (12, 13). In addition, with a high false positive rate, DNA quantitative cytology test or sequencing of circulating tumor DNA had not been optimized enough in a large-scale screening either (14, 15). Limitations also exist in the endometrial cytology test (ECT) on early screening and in the diagnosis of EC, such as a lack of enough cytopathologists and a unified diagnostic standard (16).

More and more studies on epigenetics reveal a close association between DNA methylation and the progression of cancer. Some methylation biomarkers have been applied for early detection, diagnosis, and prognosis (17). They could differentiate cancerous tissue from normal tissue with >95% accuracy in common cancers (18). DNA methylation of cytological specimens, as a minimally or non-invasive biomarker, is explored extensively in solid tumors, such as cervical cancer using liquid-based cervical scrapes, urothelial tract carcinoma using urinary samples, and lung cancer using bronchoalveolar lavage fluid samples (19–21). Compared with the benign endometrium (BE), tissue specimens of EC and precancerous lesions show significant differences in methylation levels (22–24). There is an urgent need to find a comprehensive panel of methylation biomarkers for more minimally invasive, accurate, and economical screening methods of EC.

In the present study, we aimed to propose a novel method for the screening of EC. Two hypermethylated candidate genes, namely, cysteine dioxygenase type 1 (*CDO1*) and zinc finger protein 454 (*ZNF454*), were selected in patients with EC from The Cancer Genome Atlas (TCGA) database and literature. The sensitivity (Se) and Sp of the two genes were examined on endometrial cytology. The area under the curve (AUC) of receiver operating characteristic (ROC) of methylation assays were also detected to estimate the diagnostic value for EC and AH.

## Materials and Methods

### Identification of Candidate Biomarkers

To identify candidate genes, the DNA methylation data from a total of 431 EC tissue samples and 46 corresponding adjacent normal endometrial samples were obtained from TCGA database (http://cancergenome.nih.gov/), along with the corresponding clinical information (**Supplementary Table 1**). The methylation score for each CpG in genome-wide identification and validation was represented as a β-value and normalized, and the details were described previously (25). The β-value indicated a level of DNA methylation ranging from 0 (unmethylated) to 1 (fully methylated). Different cutoff values were defined as hypomethylation (β-value: 0.3–0.25) and hypermethylation (β-value: 0.7–0.5). The sex chromosome probes were removed, and the remaining CpG sites were analyzed in 22 pairs of chromosomes. Only the top 5% of genes with differences in hypermethylation from the mean β-values of tumor (β_T_) minus the normal values (β_N_) and Δβ (β_T_-β_N_) ≥ 0.3 were selected. We focused on probes of CpG sites located closest (≤2 kb) to the upstream of the transcriptional start site (TSS) of the coding genes. If several CpG sites, we would take the average value as the methylation value of the gene. Finally, we selected two hypermethylated candidates with ≥5 remaining CpG sites in the coding gene based on relevant literature, for further validation.

Then, the two genes were further confirmed hypermethylated in EC by first-generation sequencing using randomly collected specimens of cancerous and non-cancerous tissues after hysterectomy. The process of recruitment was the same as the patients in the training set.

### Recruitment of Histological and Cytological Specimens

According to the method to calculate sample size in the diagnosis experiment (26) and the Se and Sp of candidate genes in relevant studies (22, 27, 28), a minimum of 41 cases in the malignant group and 57 cases in the benign group were required for our study (Se was taken as 0.88 and Sp was taken as 0.82 to predict results).

From June 2019 to January 2020, women with certain diagnosis, including EC, AH, or benign diseases, such as uterine myoma, adenomyosis, and uterine prolapse, underwent total hysterectomy in The First Affiliated Hospital of Xi’an Jiaotong University. Their endometrial tissues specimens obtained from surgical resections were formalin-fixed and paraffin-embedded, as a training set for histological DNA methylation analysis. The histopathological diagnosis using hematoxylin and eosin staining was confirmed by two pathologists as the reference standard. Inclusion criteria were as follows: 1) patients were at 25–90 years old and 2) patients without systemic diseases or other malignant tumors except for EC. Exclusion criteria were as follows: clinical information was incomplete or non-traceable.

As a testing set, endometrial cytological samples were prospectively collected from patients in The First Affiliated Hospital of Xi’an Jiaotong University, Tangdu Hospital of the Fourth Military Medical University, and Shaanxi Provincial Cancer Hospital with Li brushes (Xi’an Meijiajia Medical Co., 20152660054) before hysterectomy or D&C. These patients were suspected of having EC, AH, or benign diseases. Specimens were sampled by professional gynecologists after standard training. The procedure of endometrial sampling using Li Brush was shown in our previous study (16). First, the patients were placed in the lithotomy position. After conventional perineal and vaginal disinfection (iodine), the uterine cervix was exposed by a sterile speculum and the uterine depth was detected with uterine probe. Second, the sampler was put into the fundus of uterus after the brush head was hidden in the drivepipe. The drivepipe was drawn out 5 mm to show the brush, and the handle was rotated 5–10 complete circles to gather cells of the uterine corpus. Third, the drivepipe was advanced 3 mm and the handle was again rotated to gather cells from the uterine fundus. Last, the brush was removed from uterine cavity after protecting the brush head under the casing. When sampling was complete, endometrial cells were collected in the preservation solution for DNA methylation test. Our previous study showed that cytology by Li brush had a lower insufficient sample rate than did D&C (29). All of the patients were pathologically diagnosed by their tissues or cells using hematoxylin and eosin staining. The pathological results were defined as gold standard and demographic data were well documented. Inclusion criteria were the same as described above in the training set. Exclusion criteria were as follows: 1) insufficient quantity of samples for methylation test and 2) clinical information was incomplete or non-traceable.

This article was approved by the Ethics Committee of The First Affiliated Hospital of Xi’an Jiaotong University (XJTU1AF2015LSL-007). Informed consent was obtained from every participant in this study. All participants were recruited randomly.

The test criteria included Se, Sp, and accuracy. Se was defined as the probability that a patient who was diagnosed as positive (EC and AH) by the gold standard showed a positive methylation result. Sp was defined as the probability that a person who was diagnosed as negative (BE) by the gold standard had a negative result for methylation test. Accuracy was the ratio when the methylation result and cytopathologic or histopathologic diagnoses were both positive or both negative.

### Quantitative Methylation-Specific Polymerase Chain Reaction

Genomic DNA was extracted from paraffin-embedded endometrial tissue and cells specimens (from hysterectomy, D&C, and cytology) using the TIANamp Genomic DNA Kit (TIANGEN Biotech Co., DP304, Beijing, China). According to the manufacturer’s instructions, tissues were prepared in a cell suspension and digested with Proteinase K at 56°C until being dissolved. The genomic DNA was dissolved in nuclease-free water and bisulfite converted using the DNA Bisulfite Conversion Kit (TIANGEN Biotech Co., DP215, Beijing, China). Two pairs of primers were designed, allowing separate amplification of populations representing methylated and unmethylated DNA fragments. To confirm the quality and quantity of bisulfite-modified DNA, ACTB was used to normalize the DNA input, thus allowing unbiased amplification. Primers to measure the amount of the non-CpG region of ACTB in each methylation-independent assay were designed as controls. These primers were designed by Oligo 7.0 Primer Analysis software (Molecular Biology Insights, Inc., Colorado Springs, CO, USA). Polymerase chain reaction (PCR) products were amplified with the LightCycler 480 SYBR Green I Master (Roche, Indianapolis, IN, USA) and performed using ABI7500 (Applied Biosystems, Thermo Fisher Scientific Corp., Waltham, MA, USA). A 25 μl of reaction contained 5 μl of bisulfite-converted DNA, 250 nmol/L each primer, and 10 μl of Master Mix. The reactions were conducted under the following thermal profiles: 95°C for 10 min, 45 cycles consisted of denaturing at 95°C for 20 s, annealing at 60°C for 30 s. The melt curve data were generated by increasing the temperature from 60°C to 90°C and recording fluorescence. All specimens conducted triplicate testing in each gene.

The cycle threshold (Ct)–values of the two PCR reactions were compared to determine the methylation level of the site. The lower the Ct-values, the higher the gene methylation status. The ACTB was used as an internal reference gene by amplifying non-CpG sequences. The DNA methylation level was estimated as the ΔCt by using the formula: Ct (target gene) − Ct (ACTB). The distribution of the dot plots represents the methylation levels, in terms of change in Ct (ΔCt-value) of each candidate gene. We generated the best cutoff values of ΔCt from the tissues or cells to distinguish between benign and cancerous subjects. Methylation was considered as positive when ΔCt-value ≤ the cutoff value (at least two replicates).

### Statistical Analysis

Patients and tumor characteristics were tabulated. Normality test was used to determine whether the variance of the population was equal by Kolmogorov–Smirnov test, and age differences between groups as continuous variables were compared using Student’s *t*-test (two-tailed). The significant differences of methylated CpG sites between cancerous and benign specimens were analyzed using the Mann–Whitney *U*-test. In addition, the differences between EC, AH, and BE were analyzed using the Kruskal–Wallis test (*P* < 0.001). ROC curves were generated to determine the ΔCt cutoff values of candidate gene methylation. The Se, Sp, and AUC of each gene, or genetic combination, were calculated. The differences of the accuracy between the training set and the testing set were considered significant if the *P*-value of Chi-squared test was < 0.05. Data were analyzed using SPSS version 20.0 software (SPSS Inc., Chicago, IL, USA).

## Results

### Candidate Biomarkers

**Figure 1:**
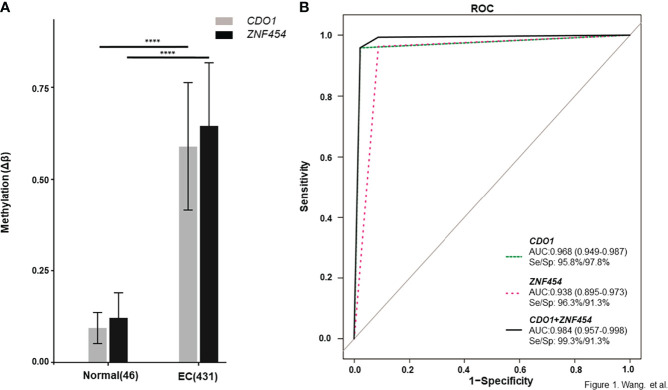
DNA methylation levels of candidate genes from TCGA database. DNA methylation levels are displayed as Δβ-values for each candidate gene. **(A)** *CDO1* and *ZNF454* methylation levels between cancerous and normal tissues. **(B)** The area under the receiver operating characteristic curve (AUC-ROC) for the DNA methylation status of *CDO1* and *ZNF454.* ****P < 0.0001.

**Figure 2:**
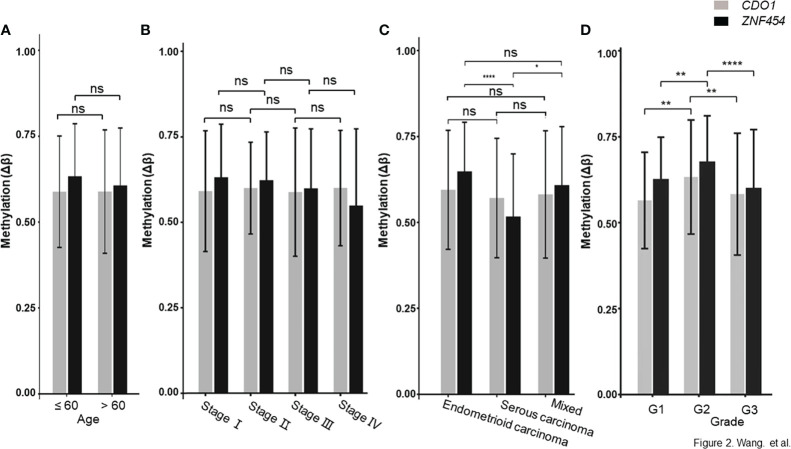
DNA methylation level between different age groups, stages and pathologic types. **(A)** *CDO1 and ZNF454* methylation levels between women aged ≤60 and >60. **(B)** *CDO1* and *ZNF454* methylation levels between different stages. **(C)** *CDO1* and *ZNF454* methylation levels between different pathological types. **(D)** *CDO1* and *ZNF454* methylation levels between different grades. ****P < 0.0001; **P < 0.01; *P < 0.05; ns, no significance.

The data that support the findings of this study are available in TCGA database. As described in the above method, a total of 75 candidate biomarkers in the top 5% were selected, of which 17 were upstream genes. Six genes with ≥5 remaining CpG sites were further analyzed and two of them, namely, *CDO1* and *ZNF454*, were picked up by referring to relevant references (22, 27, 28). Their methylation data were showed in **Supplementary Table 2**, and the methylation levels, between cancerous and normal tissues, showed a significant difference (*P* < 0.001) (**Figure 1A**). ROC curve was determined on the basis of TCGA database (**Figure 1B**). To explore further the clinical relevance of DNA methylation level, we made comparisons between different age groups, stages, pathologic parameters, and grades. We found that methylation level of *CDO1* or *ZNF454* had no statistical difference between women aged ≤60 and >60 (**Figure 2A**) and between different stages (**Figure 2B**). Methylation status of *CDO1* had no statistical difference between different pathological types. However, *ZNF454* had a significant difference between endometrioid carcinoma and serous carcinoma (*P* < 0.001) and between serous carcinoma and mixed carcinoma (*P* < 0.05) (**Figure 2C**). In addition, methylation level of *CDO1* or *ZNF454* had statistical difference between different grades (*P* < 0.01) (**Figure 2D**).

Next, the Se, Sp, and accuracy of these two genes were calculated in the initial verification using first-generation sequencing (Sanger sequencing results were partially shown in **Supplementary Figures 1** **,** **2**). Thirty-eight individual endometrial tissues were randomly collected from surgical resections in The First Affiliated Hospital of Xi’an Jiaotong University, including 25 cancerous and 13 normal tissues (**Table 1**). Se and Sp levels of *CDO1* were 92.00% and 92.31%, respectively. Se and Sp levels of *ZNF454* were 88.00% and 100.00%, respectively. In addition, their diagnostic accuracy levels were both above 92%. Therefore, the two genes, namely, *CDO1* and *ZNF454*, were determined for follow-up verification in training set.

**Table 1:** Se, Sp, and accuracy of DNA methylation of *CDO1* and *ZNF454* using first-generation sequencing.

Gene	True positive	False positive	Se (%)^a^	Sp (%)^b^	Accuracy (%)
*CDO1*	23/25	1/13	92.00	92.31	92.11
*ZNF454*	22/25	0/13	88.00	100.00	92.11

a Se, sensitivity.

b Sp, specificity.

### Verification of Candidate Genes in Histological Specimens (Training Set)

A total of 103 specimens (21 EC, 20 AH, 41 BE, and 21 adjacent benign endometrial tissues) were included in the training set. Demographics related to the clinical samples were shown in **Supplementary Table 3**. There were 20 endometrioid carcinoma and one mucinous carcinoma among 21 EC. To assess methylation differences between malignant and BE, EC and AH were included in the malignant group, with a total of 41 patients. They both required early treatment and management in clinical. From the perspective of screening, we did not distinguish EC and AH respectively. BE and adjacent benign endometrial tissues were included in benign group, with a total of 62 patients. Women in malignant group were aged from 28 to 66 years (47.10 ± 8.22 years) and those in benign group were aged from 30 to 66 years (49.63 ± 7.54 years). Age was not significantly different between the two groups (P = 0.116).

Primer sequences for qMSP analysis were designed as **Supplementary Table 4**. To determine the amplification efficiency of primers, we used methylated primers to amplify the methylated or the unmethylated templates. As shown in **Supplementary Figure 3A**, the template was well amplified when the concentration of methylated template was only 1 × 10^2^ copies/μl, whereas the unmethylated template had very low amplification even the concentration of unmethylated template reached 1 × 10^5^ copies/μl. **Supplementary Figure 3B** shows the representative curves for 100% methylated DNA and fully unmethylated DNA along with different dilution of methylated and unmethylated DNA. The melting temperature (Tm) of the unmethylated template was lower than that of the methylated template in the melt curve (**Supplementary Figure 3C**). At 86.61°C, the methylated primers specifically amplified the methylated template.

**Figure 3:**
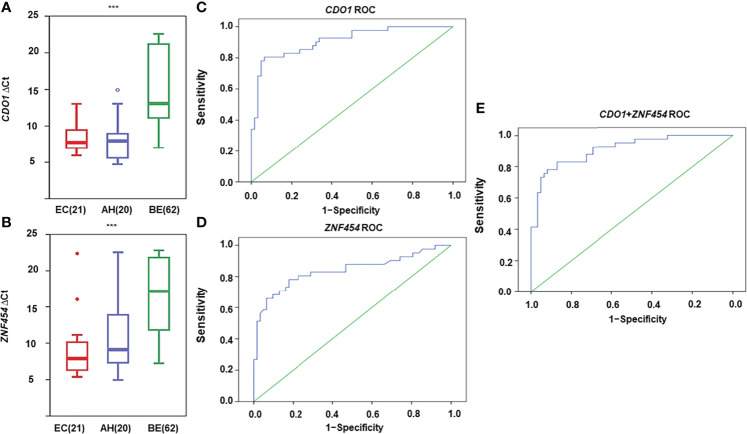
DNA methylation levels for candidate genes detected by quantitative methylation-specific polymerase chain reaction (qMSP) in histological specimens. DNA methylation levels are displayed as the difference in cycle threshold (ΔCt)–values for **(A)** *CDO1* and **(B)** *ZNF454*. Horizontal bars in the middle indicate the average methylation levels. The area under the receiver operating characteristic curve (AUC-ROC) for the DNA methylation status of **(C)** *CDO1*, **(D)** *ZNF454*, and **(E)** combination of the two genes. ***P < 0.001.

As shown in **Figures 3A, B**, the mean methylation levels of *CDO1* and *ZNF454* in EC, AH, and BE were significantly different (*P* < 0.001). By using ROC curve and AUC analysis methods, the optimal cutoff values of ΔCt of *CDO1/ZNF454* were 9.8/11.4 when the Youden index (Se + Sp − 1) reached the maximum in the ROC curves. At the thresholds, in the malignant group, 33 cases of *CDO1* methylation were positive, and eight cases were negative. In the benign group, four cases were positive and 58 cases were negative. Thus, the diagnostic Se and Sp were 80.49% and 93.55%, respectively. AUC was 0.910 (95% CI: 0.851–0.968) (**Figure 3C**). Whereas 32 cases of *ZNF454* methylation were positive and nine cases were negative in the malignant group, 11 were positive and 51 were negative in the benign group. The Se and Sp levels of *ZNF454* were 78.05% and 82.26%, respectively. AUC was 0.838 (95% CI: 0.750–0.925) (**Figure 3D**). When the two genes were combined, the Se and Sp levels were 92.68% and 82.26%, respectively. AUC was 0.911 (95% CI: 0.854–0.968) (**Figure 3** **E**).

### Methylation Assays of Candidate Genes in Cytological Specimens (Testing Set)

A total of 174 cytological specimens were collected, of which 25 cases that did not have a sufficient number of endometrial cells for the methylation test and 29 cases that lacked detailed pathological information were successively excluded. The degree of satisfaction of sampling for the methylation test was 85.63% (149 of 174) and 120 cytological specimens were left in an independent testing set (42 EC, 2 AH, and 76 BE). Their demographics were shown in **Supplementary Table 3**, and one of the patients did not undergo hysterectomy for financial reasons, so her stage of EC was unknown. Among 42 EC, there were 38 endometrioid carcinoma, two mucinous carcinoma, one serous carcinoma, and one mixed serous and endometrioid carcinoma. Women in the malignant group (EC and AH) were aged from 28 to 74 years (51.48 ± 9.67 years), and those in the benign group were aged from 23 to 77 years (48.82 ± 10.11 years). Age was not significantly different between the two groups (*P* = 0.161).

**Figure 4:**
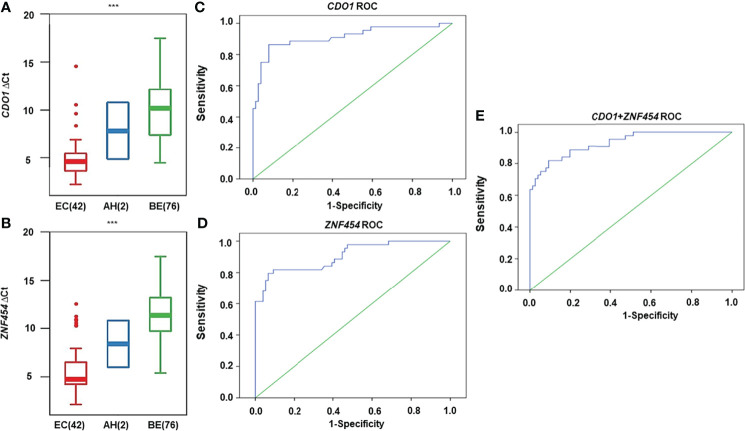
DNA methylation levels for candidate genes detected by quantitative methylation-specific polymerase chain reaction (qMSP) in cytological specimens. DNA methylation levels are displayed as the difference in cycle threshold (ΔCt)–values for **(A)** *CDO1* and **(B)** *ZNF454*. Horizontal bars in the middle indicate the average methylation levels. The area under the receiver operating characteristic curve (AUC-ROC) for the DNA methylation status of **(C)** *CDO1*, **(D)** *ZNF454*, and **(E)** combination of the two genes. ***P < 0.001.

As shown in **Figures 4A, B**, *CDO1* and *ZNF454* methylation levels were significantly different among EC, AH, and BE (*P* < 0.001). When the Youden index reached the maximum in the ROC curves, the cutoff ΔCt-values of *CDO1*/*ZNF454* were 6.0/7.1. Thus, in the malignant group, 38 cases of *CDO1* methylation were positive, and six cases were negative. In the benign group, seven cases were positive, and 69 cases were negative. Se level was 86.36%, and Sp level was 90.79%. AUC was 0.911 (95% CI: 0.850–0.973) (**Figure 4C**). Thirty-five cases of *ZNF454* methylation were positive, and nine cases were negative in the malignant group. Five cases of *ZNF454* methylation were positive, and 71 cases were negative in the benign group. Se and Sp levels were 79.55% and 93.42%, respectively. AUC was 0.905 (95% CI: 0.848–0.963) (**Figure 4D**). When the two genes were combined, Se and Sp levels were 90.91% and 86.84%, respectively. AUC increased to 0.931 (95% CI: 0.885–0.976) (**Figure 4E**). Compared with the indicators of individual genes, the Se and AUC were best but Sp was slightly lower.

### Comparison of the Accuracy of Histological and Cytological Methylation

The diagnostic accuracy of the genes was calculated in the detection of EC and AH from the training set and the testing set. The diagnostic accuracy of *CDO1*/*ZNF454* methylation had no statistical difference between cytological specimens and histological specimens (*P* = 0.847/*P* = 0.108). When the two genes were combined, the accuracy had no significant difference (*P* = 0.665) (**Table 2**). The diagnostic accuracy of the candidate genes methylation in cytological specimens was not worse than that in the histological samples.

**Table 2:** Comparison of gene methylation level on diagnostic accuracy.

	Accuracy (*CDO1*) (%)	Accuracy (*ZNF454*) (%)	Accuracy (*CDO1* + *ZNF454*) (%)
Histological (n = 103)	88.35	80.58	86.41
Cytological (n = 120)	89.17	88.33	88.33
χ^2^	0.037	2.577	0.187
*p*	0.847	0.108	0.665

**Figure 5:**
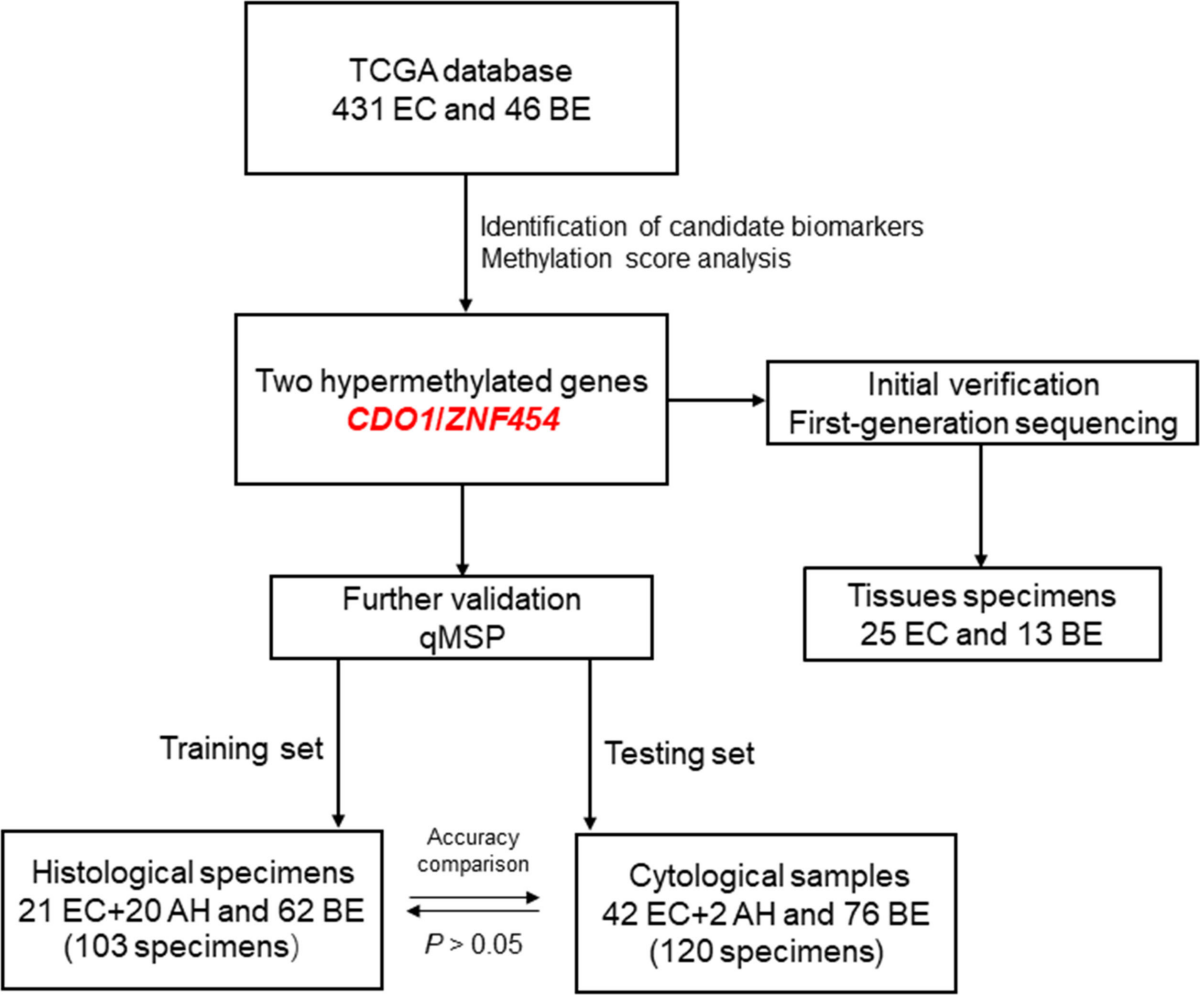
The flow chart of this study. EC, endometrial cancer; BE, benign endometrium; AH, atypical hyperplasia; qMSP, quantitative methylation-specific polymerase chain reaction.

The flow chart of this study is shown in **Figure 5**.

## Discussion

We proved a relatively high Se, Sp, and AUC of *CDO1* or *ZNF454* methylation test in the cytological detection of EC and AH. When the two candidate genes were combined, clinical performance was better because of lower missed diagnosed rate (1 − Se) and higher AUC. Diagnostic accuracy in cytological specimens had no significant difference compared with that in histological ones. It was of great help in scenarios of limited availability or quality of tissue.

Compared with relevant studies conducted by Wentzensen’s team (30, 31), our research showed some advantages. First, our analysis demonstrated the Se and Sp in detail, with higher AUCs and a larger sample size. A total of 120 cytological specimens were collected for the diagnostic test and the methylation biomarker panel obtained an AUC of 0.931, with a 90.91% Se and an 86.84% Sp. Although Wentzensen et al. estimated an Sp of only > 50% and a summary AUC of 0.85 in 38 EC and 37 BE specimens, they found that among women with carcinoma, median methylation levels of most candidate genes in frozen carcinoma tissues and cell specimens seemed to be similar, but the *P*-value was not calculated (31). In their follow-up study, the methylation levels of candidate genes were compared in 38 EC and 28 BE. The best AUC for a single gene was 0.90. However, the Se and Sp were not calculated (30). A systematic review and meta-analysis including 22 studies showed that the pooled Se and Sp levels of DNA methylation in sporadic EC were 93% and 48%, respectively, and the AUC of summary ROC was 0.8834 (32). Apparently, we seem to have a better Sp and AUC using only two candidate genes. Second, *CDO1* and *ZNF454* were identified from the TCGA database and validated through a rigorous selection process, as described in Materials and Methods. *CDO1*, as a tumor suppressor gene, was also reported hypermethylated in cervical scrapings of patients with EC by Huang et al. (22, 33). *ZNF454* may be a tumor-suppressor gene with potential use as a prognostic indicator (27), but its methylation site was not previously reported in EC. Our study found that *ZNF454*, with relatively high Se, Sp, and AUC, was also a promising biomarker on EC screening. Although Wentzensen et al. investigated the diagnostic potential of seven genes (*ADCYAP1*, *ASCL2*, *CDH13*, *HS3ST2*, *HTR1B*, *MME*, and *NPY*), they identified hypermethylated in EC (31) and five other genes (*GTF2A1*, *HAAO*, *HOXA9*, *HSP2A*, and *RASSF1*) observed by other research studies (30). Third, endometrial sampling was performed by Li brushes in our study but Tao brushes (Cook Medical, Bloomington, IN, USA) in research studies of Wentzensen et al. Although our comparative analysis showed a high diagnostic accordance between the Li brush and Tao brush in evaluating EC and AH, Tao brush is more expensive and rarely available in China, which increases the screening cost (29). Considering social and economic benefits, Li brush may be a better choice in the screening program.

Our research provides a minimally invasive method for EC and AH screening. Considering the low EC prevalence, traditional invasive D&C to obtain endometrial tissue is redundant and unnecessary procedure in screening of large population (34). For high-risk population, such as those with Lynch syndrome and obesity (6), endometrial brush sampling brings less discomfort than invasive D&C (16). Cytological DNA methylation test reduces the workload of pathologists, which is of great value especially in countries with a shortage of cytopathologists. As a preliminary screening test, when its positive result occurs, gynecologists perform a further biopsy for a definite diagnosis.

Some studies proposed other minimally invasive and indirect methods to screen EC through the DNA methylation analysis, such as cervical scrapings, intravaginal tampons, and urine fractions (22, 30, 33, 35–37). Huang et al. revealed a Se level of between 87.0% and 91.8% and a Sp level of between 80.0% and 95.5% for EC detection by a three-gene panel in cervical scrapes (22, 33). Chang et al. reported a Se level of 83%–90% and a Sp level of 69%–88% using cervical scrapings in the detection of EC (36). In studies using intravaginal tampons, although Sp reached 100%, Se was only 37%–40%. The maximum AUC to distinguish malignant endometrial tumors was only 0.82 (35). Considering that only a tiny number of abnormal cells exfoliated from the uterine cavity could be captured in the early stage of EC, the detection with cervical scrapings during the Papanicolaou test or vaginal pool samples in tampons was opportunistic (38). DNA methylation analysis in urine provided an alternative for the detection of EC. In all urine fractions (full void, sediment, and supernatant), three DNA methylation markers (*GHSR*, *SST*, and *ZIC1*) showed increased methylation levels in patients with EC as compared to controls, with AUC values ranging from 0.86 to 0.95 (37). The results seemed to be encouraging, but more exploration was needed. In our study, endometrial cells were collected directly from the uterine cavity, which achieved rather satisfactory results.

Despite some encouraging results, our research still had several limitations. We had not stratified our analysis based on the body mass index, categories of pathology, grades, or clinical developments (AH to EC). These factors could have been associated with distinct DNA methylation signatures (24, 39, 40). **Figures 2C, D** in the current study also suggested that DNA methylation status might be related to histopathology. As an extension of the current work, further validation in stratified, population-based studies should be conducted to determine more applicable cutoff values of DNA methylation of better biomarkers.

## Conclusion

We conducted a series of identification and verification work to find DNA methylated biomarkers associated with abnormal endometrium (including EC and AH). As a preliminary screening test, *CDO1* and *ZNF454* methylation analysis in cytological specimens improved the screening procedures of EC and AH. It provided a valuable and minimally invasive method to distinguish women with EC and AH from the vast majority of women without neoplastic lesions.

## Data Availability Statement

The original contributions presented in the study are included in the article/**Supplementary Material**. Further inquiries can be directed to the corresponding authors.

## Ethics Statement

The studies involving human participants were reviewed and approved by the Ethics Committee of the First Affiliated Hospital of Xi’an Jiaotong University. The patients/participants provided their written informed consent to participate in this study.

## Author Contributions

Conceptualization: LW, LLZ, and QL. Data curation, formal analysis, investigation, methodology, and visualization: LW, LD, JX, LG, YW, KW, WJ, LBZ, XF, KZ, MG and YZ. Project administration and resources: LW, LD, LG, YW, LBZ, and QL. Supervision and validation: LW, LD, JX, KW, and QL. Writing—original draft: LW, LD, and QL. Writing—review and editing: All authors. All authors contributed to the article and approved the submitted version.

## Funding

This study was funded by the Clinical Research Award of The First Affiliated Hospital of Xi’an Jiaotong University (No. XJTU1AF-CRF-2019-002) and the Key Research and Development Project of Shaanxi Provincial Science and Technology Department (No. 2019QYPY-138). The sponsors have no involvement in study design, data collection, analysis, and interpretation or in writing of the manuscript.

## Conflict of Interest

LD, JX, KW, and LLZ were employed by Wuhan ammunition Life-Tech Co., Ltd.

The remaining authors declare that the research was conducted in the absence of any commercial or financial relationships that could be construed as a potential conflict of interest.

## Publisher’s Note

All claims expressed in this article are solely those of the authors and do not necessarily represent those of their affiliated organizations, or those of the publisher, the editors and the reviewers. Any product that may be evaluated in this article, or claim that may be made by its manufacturer, is not guaranteed or endorsed by the publisher.

## Abbreviations

AH, atypical hyperplasia; AUC, area under the curve; BE, benign endometrium; *CDO1*, cysteine dioxygenase type 1; Ct, cycle threshold; D&C, dilation and curettage; EC, endometrial cancer; ECT, endometrial cytology test; PCR, polymerase chain reaction; qMSP, quantitative methylation-specific polymerase chain reaction; ROC, receiver operating characteristic; Se, sensitivity; Sp, specificity; TCGA, The Cancer Genome Atlas; TSS, transcriptional start site; *ZNF454*, zinc finger protein 454.
