# Supplementary figures and images for: Hypermethylated CDO1 and ZNF454 in Cytological Specimens as Screening Biomarkers for Endometrial Cancer
Source: Front Oncol. 2022 Apr 28;12:714663. doi: 10.3389/fonc.2022.714663 (PMC9095965; doi:10.3389/fonc.2022.714663)

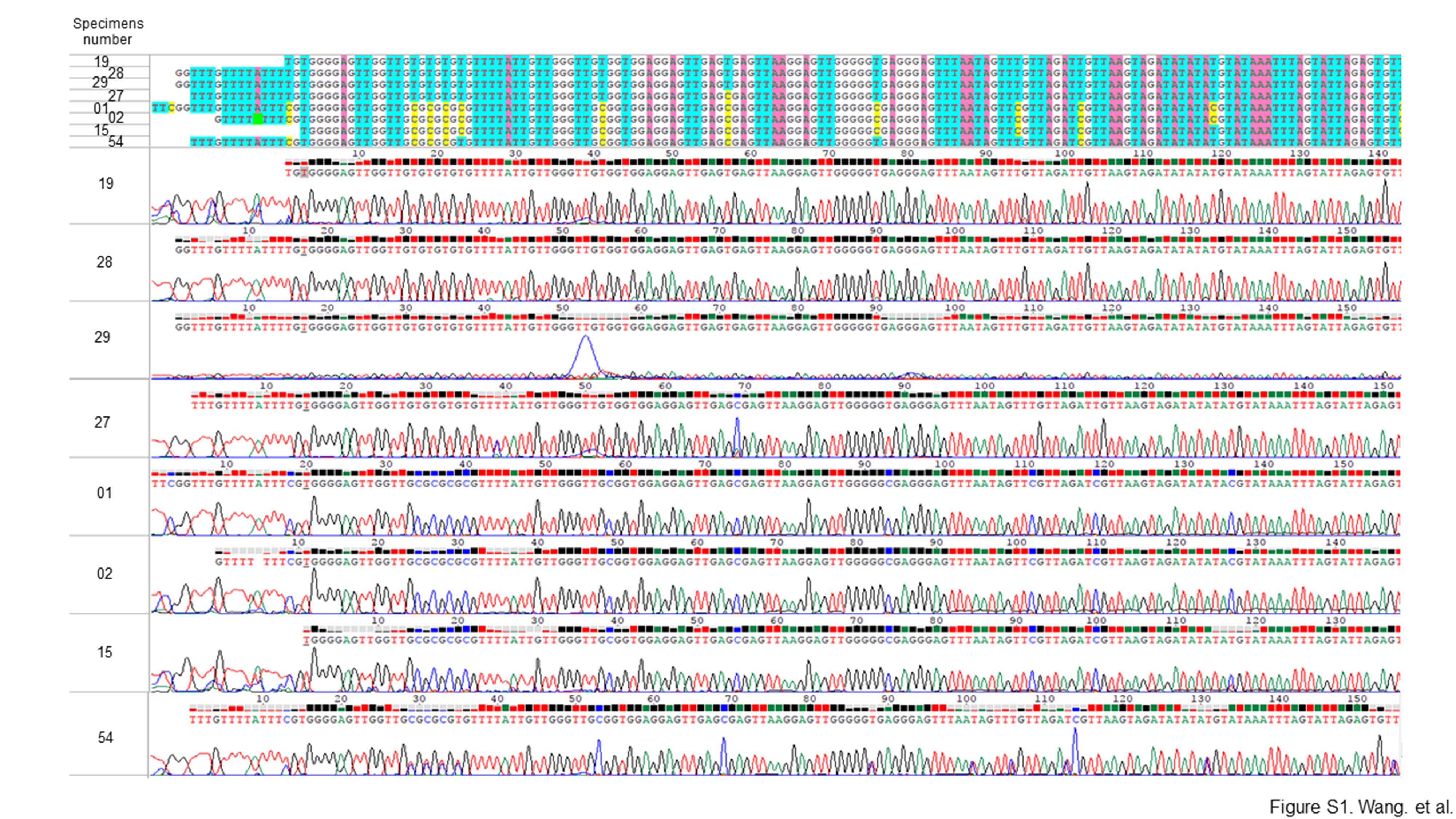

Supplement: Supplementary file 1 [file Image_1.tif]

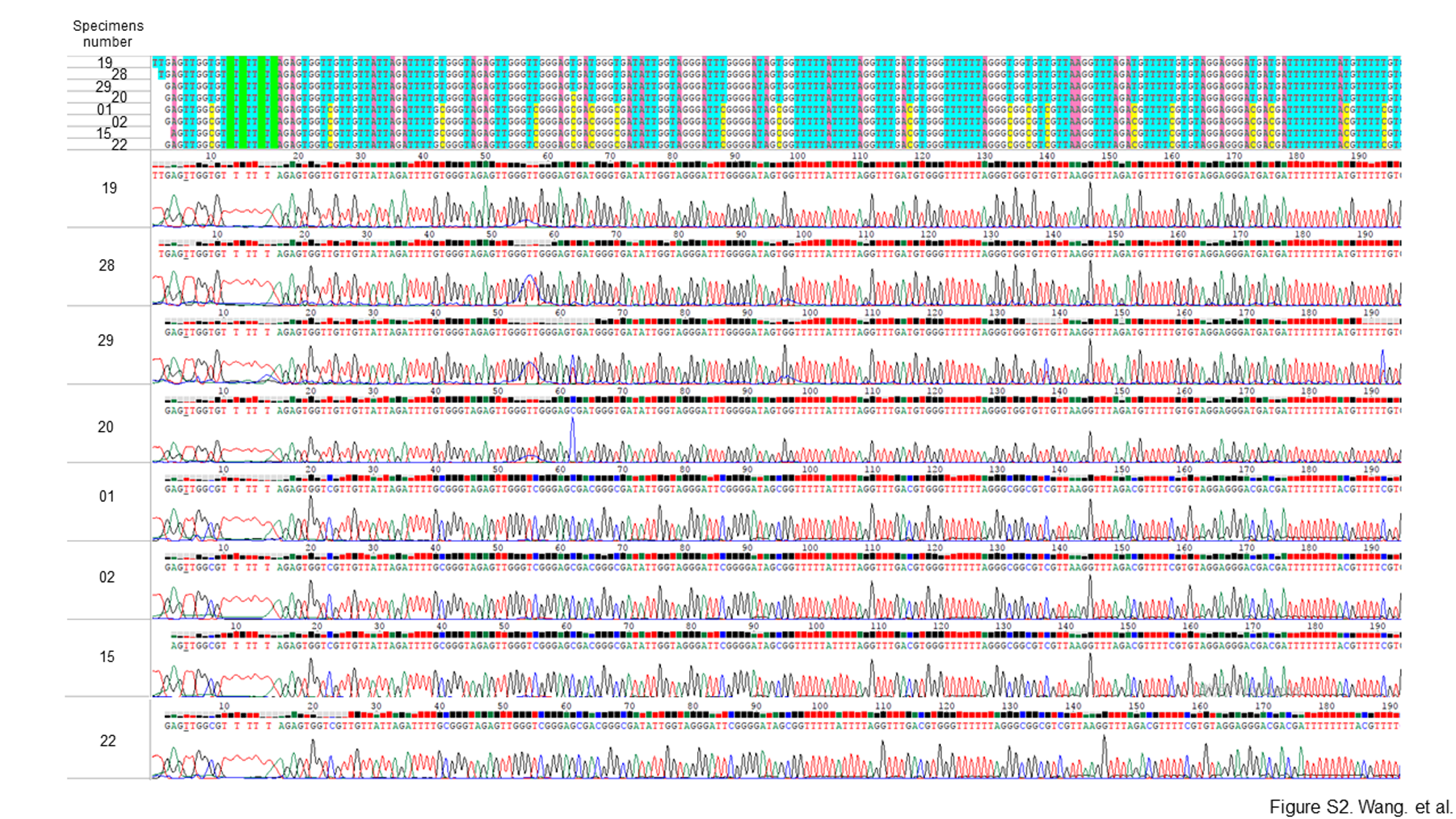

Supplement: Supplementary file 2 [file Image_2.tif]

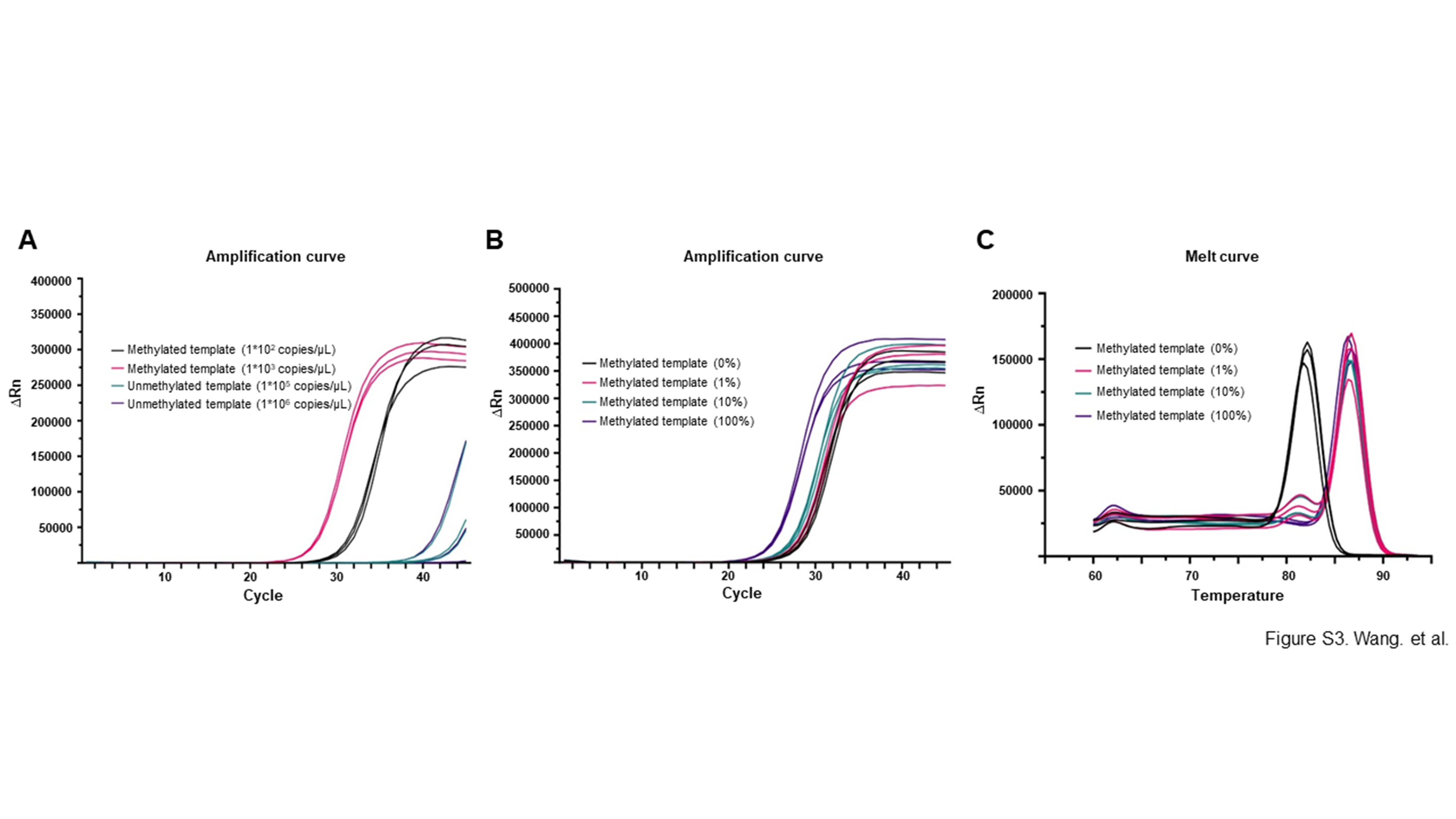

Supplement: Supplementary file 3 [file Image_3.tif]
