# Supplementary material for: Hypermethylated CDO1 and ZNF454 in Cytological Specimens as Screening Biomarkers for Endometrial Cancer
Source: Front Oncol. 2022 Apr 28;12:714663. doi: 10.3389/fonc.2022.714663 (PMC9095965; doi:10.3389/fonc.2022.714663)
Supplement: Supplementary file 4 [file DataSheet_1.docx]

Supplementary Material

**Figure S1.** First-generation sequencing results of *CDO1* (only eight specimens were displayed). Among them, specimen 19/28/29/27 were considered as negative and specimen 01/02/15/54 were positive based on the results of quantitative methylation-specific polymerase chain reaction (qMSP). Cytosine was retained (yellow colored) after being bisulfite converted, indicating that the site was methylated. So, specimen 19/28/29/27 were unmethylated and 01/02/15/54 were methylated in the results of Sanger sequencing. Among them, specimen 01/02 were fully methylated, while 15/54 were partially methylated, which meant that the partially methylated genes were amplified by qMSP.

**Figure S2.** First-generation sequencing results of *ZNF154* (only eight specimens were displayed). Among them, specimen 19/28/29/20 were considered as negative and specimen 01/02/15/22 were positive based on the results of quantitative methylation-specific polymerase chain reaction (qMSP). Cytosine was retained (yellow colored) after being bisulfite converted, indicating that the site was methylated. So, specimen 19/28/29 were unmethylated and 01/02/15/22/20 were methylated in the results of the Sanger sequencing.

**Figure S3.** Representative amplification curve and melt curve for quantitative methylation-specific polymerase chain reaction (qMSP). (**A**) Amplification curve of the methylated primers. (**B**) Amplification curve of fully unmethylated DNA and 100% methylated DNA along with different dilution of methylated and unmethylated DNA. (**C**) Melting temperature (Tm) curve of fully unmethylated DNA and 100% methylated DNA along with different dilution of methylated and unmethylated DNA.

**Supplementary Table 1.** Clinical Information from TCGA.

| Variable | *n* |
| --- | --- |
| Gender |  |
| Female | 431 |
| Sample type |  |
| Cancer | 431 |
| Adjacent benign endometrium | 46 |
| Median Age(range) | 64(31-90) |
| Clinical stage(%) |  |
| Stage I | 263(61.02%) |
| Stage II | 43(9.98%) |
| Stage III | 101(23.43%) |
| Stage IV | 24(5.57%) |
| Histological type(%) |  |
| Endometrioid carcinoma | 312(72.39%) |
| Mixed serous and endometrioid | 21(4.87%) |
| Serous carcinoma | 98(22.74%) |
| Histological grade(%) |  |
| G1 | 63(14.62%) |
| G2 | 91(21.11%) |
| G3 | 266(61.72%) |
| Unknow | 11(2.55%) |

**Supplementary Table 2.** Methylation level of *CDO1* and *ZNF454.*

| Gene | Methylation Site Probe | Chr | Position | Mean β_N_ (SD^a^) | Mean β_T_(SD^a^) | Δβ |
| --- | --- | --- | --- | --- | --- | --- |
| *CDO1* | cg02792792 | 5 | 115180319 | 0.125(0.067) | 0.647(0.166) | 0.522 |
|  | cg08516516 | 5 | 115180391 | 0.021(0.037) | 0.599(0.205) | 0.578 |
|  | cg11036833 | 5 | 115180393 | 0.032(0.080) | 0.606(0.170) | 0.574 |
|  | cg14470895 | 5 | 115180285 | 0.041(0.047) | 0.704(0.189) | 0.664 |
|  | cg16707405 | 5 | 115180330 | 0.041(0.034) | 0.690(0.198) | 0.649 |
|  | cg23180938 | 5 | 115180225 | 0.028(0.056) | 0.704(0.196) | 0.676 |
|  | cg12880658 | 5 | 115180312 | 0.125(0.048) | 0.669(0.177) | 0.543 |
|  | cg16265906 | 5 | 115180384 | 0.098(0.044) | 0.544(0.175) | 0.446 |
| Average |  |  |  | 0.077(0.042) | 0.622(0.174) | 0.545 |
| *ZNF454* | cg02165355 | 5 | 178300677 | 0.118(0.083) | 0.661(0.168) | 0.543 |
|  | cg03234732 | 5 | 178300729 | 0.062(0.053) | 0.658(0.195) | 0.596 |
|  | cg03355526 | 5 | 178301021 | 0.181(0.084) | 0.567(0.137) | 0.386 |
|  | cg10575261 | 5 | 178300766 | 0.056(0.051) | 0.524(0.192) | 0.468 |
|  | cg10902717 | 5 | 178301226 | 0.106(0.065) | 0.634(0.180) | 0.528 |
|  | cg16536329 | 5 | 178300791 | 0.140(0.070) | 0.632(0.165) | 0.492 |
|  | cg20778451 | 5 | 178300859 | 0.082(0.072) | 0.604(0.175) | 0.522 |
|  | cg23037403 | 5 | 178300811 | 0.133(0.061) | 0.613(0.169) | 0.48 |
|  | cg24843380 | 5 | 178300789 | 0.032(0.052) | 0.757(0.224) | 0.725 |
|  | cg17840719 | 5 | 178300433 | 0.057(0.106) | 0.805(0.188) | 0.748 |
| Average |  |  |  | 0.094(0.065) | 0.641(0.163) | 0.546 |

^a^ SD, standard deviation.

**Supplementary Table 3.** Demographics related to the clinical samples.

| Variable | Histological specimens | Cytological specimens |
| --- | --- | --- |
| **Malignant group** |  |  |
| Case number | 21 EC+20 AH | 42 EC+2 AH |
| Age(yrs) ± SD^a^ | 47.10 ± 8.22 | 51.48 ± 9.67 |
| Clinical stage(%) |  |  |
| Stage I | 21(100%) | 32(76.19%) |
| Stage II | 0(0%) | 7(16.67%) |
| Stage III | 0(0%) | 1(2.38%) |
| Stage IV | 0(0%) | 1(2.38%) |
| Unknow | 0(0%) | 1(2.38%) |
| Histological grade(%) |  |  |
| G1 | 17(80.95%) | 10(23.81%) |
| G2 | 2(9.52%) | 28(66.67%) |
| G3 | 2(9.52%) | 4(9.52%) |
| Unknow | 0(0%) | 0(0%) |
| **Benign group** |  |  |
| Case number | 62 | 76 |
| Median Age(range) | 49.63 ± 7.54 | 48.82 ± 10.11 |

^a^ SD, standard deviation.

**Supplementary Table 4.** Primer sequences for qMSP analysis.

| Gene name | Primers Sequences |
| --- | --- |
| *CDO1* | F: GGCGAGGGAGTTTAATAGTTC |
|  | R: GACGACATCCTTACGTTTCG |
|  | P: CTAAATTTATACGTATATATC |
| *ZNF454* | F: GTAATGAGCGCGTTGAGTTC |
|  | R: GCCTACGAAAACAAAACACG |
|  | P: CCGACGCTTTAAACGCGAA |
| *ACTB* | F: GGGATATGTAGAAAGTGTAAAG |
|  | R: CACAATAAATCTAAACAAACTCC |
|  | P: CCCAACACACTTAACCA |

F, forward; R, reversed; P, probe.
